# Interventions for renal vasculitis in adults. A systematic review

**DOI:** 10.1186/1471-2369-11-12

**Published:** 2010-06-24

**Authors:** Giles D Walters, Narelle S Willis, Jonathan C Craig

**Affiliations:** 1 Senior Staff Specialist, The Canberra Hospital, Canberra, Australia. Hon. Senior Lecturer, Australian National University Medical School, Canberra, Australia; 2 Managing Editor, Cochrane Renal Group, Centre for Kidney Research, Westmead, Australia; 3 The Children's Hospital at Westmead, Sydney, Australia School of Public Health, University of Sydney, Sydney, Australia

## Abstract

**Background:**

Renal vasculitis presents as rapidly progressive glomerulonephritis and comprises of a group of conditions characterised by acute kidney failure, haematuria and proteinuria. Treatment of these conditions involves the use of steroid and non-steroid agents with or without adjunctive plasma exchange. Although immunosuppression has been successful, many questions remain unanswered in terms of dose and duration of therapy, the use of plasma exchange and the role of new therapies. This systematic review was conducted to determine the benefits and harms of any intervention for the treatment of renal vasculitis in adults.

**Methods:**

We searched the Cochrane Central Register of Controlled Trials, the Cochrane Renal Group Specialised Register, MEDLINE and EMBASE to June 2009. Randomised controlled trials investigating any intervention for the treatment of adults were included. Two authors independently assessed study quality and extracted data. Statistical analyses were performed using a random effects model and results expressed as risk ratio with 95% confidence intervals for dichotomous outcomes or mean difference for continuous outcomes.

**Results:**

Twenty two studies (1674 patients) were included. Plasma exchange as adjunctive therapy significantly reduces the risk of end-stage kidney disease at 12 months (five studies: RR 0.47, CI 0.30 to 0.75). Four studies compared the use of pulse and continuous administration of cyclophosphamide. Remission rates were equivalent but pulse treatment causes an increased risk of relapse (4 studies: RR 1.79, CI 1.11 to 2.87) compared with continuous cyclophosphamide. Azathioprine has equivalent efficacy as a maintenance agent to cyclophosphamide with fewer episodes of leukopenia. Mycophenolate mofetil may be equivalent to cyclophosphamide as an induction agent but resulted in a higher relapse rate when tested against Azathioprine in remission maintenance. Rituximab is an effective remission induction agent. Methotrexate or Leflunomide are potential choices in remission maintenance therapy. Oral co-trimoxazole did not reduce relapses significantly in Wegener's granulomatosis.

**Conclusions:**

Plasma exchange is effective in patients with severe ARF secondary to vasculitis. Pulse cyclophosphamide results in an increased risk of relapse when compared to continuous oral use but a reduced total dose. Whilst cyclophosphamide is standard induction treatment, rituximab and mycophenolate mofetil are also effective. Azathioprine, methotrexate and leflunomide are effective as maintenance therapy. Further studies are required to more clearly delineate the appropriate place of newer agents within an evidence-based therapeutic strategy.

## Background

Renal vasculitis presents as rapidly progressive glomerulonephritis (RPGN) which comprises a group of conditions characterised by acute kidney failure (AKF), haematuria and proteinuria. Histological examination of the kidney reveals severe inflammation in the form of crescent formation, glomerular necrosis and vasculitis of small and medium sized vessels within the kidney. These conditions include the anti-neutrophil cytoplasmic antibody (ANCA) associated vasculitides (AAV), anti-glomerular basement membrane (anti-GBM) disease and idiopathic RPGN [1]. AAV are generally small vessel vasculitides and include Wegener's granulomatosis, microscopic polyarteritis and renal limited vasculitis [2,3]. Evidence increasingly points to the pathogenicity of ANCA [4]. Other conditions also cause vasculitis in the kidney such as Henoch Schonlein Purpura and cryoglobulinaemia resulting in immune deposits visible on electron microscopic examination of renal tissue. The treatment of Goodpasture's disease and other forms of RPGN with granular immune deposits have an entirely separate pathogenesis to the pauci-immune (no immune deposits) forms of the disease and will not be addressed in this review.

The treatment of systemic and renal vasculitis involves the use of steroids in combination with other non-steroid agents, most commonly, cyclophosphamide (CPA) [5,1] to induce remission of disease. In the presence of kidney failure, plasma exchange is often used as an adjunct to pharmacological treatment [6-8]. Once remission is achieved, treatment is scaled back to maintenance therapy with lower doses of steroids and CPA is replaced by a less potent immunosuppressive, such as azathioprine (AZA). Co-trimoxazole has been used in Wegener's granulomatosis mainly to prevent the occurrence of pneumocystis infection, upper respiratory tract infection and subsequent relapse of disease. Various guidelines are available which summarise available treatment options and some of the evidence for their use[9-11]

These treatments are well established but many questions remain unanswered [12]. Optimal agent, dose, duration, route and frequency of treatment are uncertain. CPA can be given as a daily oral dose or as intermittent oral or intravenous doses. Intravenous regimens tend to give a lower total dose and have fewer side effects, but may later give a higher rate of relapse [13]. Treatment may include intravenous methylprednisolone or plasma exchange but their place in therapy is debated [14,15]. Other therapies including Mycophenolate mofetil (MMF), anti-TNF therapy, leflunomide, methotrexate, anti-CD52 therapy, B cell depletion therapy and intravenous immunoglobulin have been suggested [16-18] but their place is not yet clear.

A previous version of the review has been published in the Cochrane library[19]. This did not include the recent data on the use of pulse cyclophosphamide[20], mycophenolate mofetil[21], leflunomide[22], methotrexate [23], rituximab[24,25] and etanercept[26]. The most notable of these is the data on pulse versus continuous cyclophosphamide and its contribution to the current debate on whether a reduction in total dose of cyclophosphamide will increase the rate of relapse. This question cannot currently be answered by the randomised controlled trials. This systematic review data is currently the best available evidence to contribute to that debate.

## Methods

### Objectives

To evaluate the benefits and harms of any intervention used for the treatment of renal vasculitis in adults.

### Studies

All RCTs and quasi-RCTs looking at any intervention used for the treatment of renal vasculitis in adults. Quasi-RCTs were included in the protocol because we expected few RCTs to be identified. Foreign language studies were not excluded.

### Participants

All adults suffering from an episode of AKF and/or proteinuria and haematuria with a kidney biopsy showing severe acute glomerulonephritis with crescents, glomerular necrosis or other histological evidence of vasculitis. AKF was as defined by the included studies.

### Outcome measures

Mortality, kidney function, need for renal replacement therapy (RRT), number of patients relapsing, adverse effects of each intervention.

### Search methods

Relevant studies were obtained form the following sources (see Appendix 1 for more detail):

1. The Cochrane Renal Group's specialised register and the Cochrane Central Register of Controlled Trials (CENTRAL) in *The Cochrane Library*, (Issue 1, 2008).

2. MEDLINE (1966 to July 2009) using the optimally sensitive strategy developed for the Cochrane Collaboration for the identification of RCTs ([27])

3. EMBASE (1980 to July 2009) using a search strategy adapted from that developed for the Cochrane Collaboration for the identification of RCTs ([28]

4. Reference lists of nephrology textbooks, review articles and relevant studies.

### Included and excluded studies

The review was undertaken by three authors (GW, NW, JC). The titles and abstracts were screened independently by GW and NW who independently assessed abstracts. Disagreements were resolved in consultation with JC.

### Study quality

The quality of studies to be included were assessed independently by GW and NW without blinding to authorship or journal of publication using the check list designed by the Cochrane Renal Group. Discrepancies were resolved in discussion with JC. The quality items to be assessed are allocation concealment, blinding, intention to treat analysis, and completeness of follow-up. Blinding was assessed for investigators, participants, outcome assessors and data analysis.

### Statistical assessment

For dichotomous outcomes results were expressed as a risk ratio (RR) with 95% confidence intervals (CI). Data were pooled using the random effects model but the fixed effects model were also analysed to ensure robustness of the model chosen and susceptibility to outliers. Where continuous scales of measurement were used to assess the effects of treatment, the mean difference (MD) was used, or the standardised mean difference (SMD) if necessary. Heterogeneity was analysed using χ^2 ^test on N-1 degrees of freedom, with an α a of 0.05 used for statistical significance and with the I^2 ^test (Higgins 2003). I^2 ^values of 25%, 50% and 75% correspond to low, medium and high levels of heterogeneity.

The summary measure data were translated into number needed to treat (NNT) and number needed to harm (NNH) for the observed overall baseline risks. Adverse effects were tabulated and assessed with descriptive techniques.

## Results

### Description of studies

**Figure 1:**
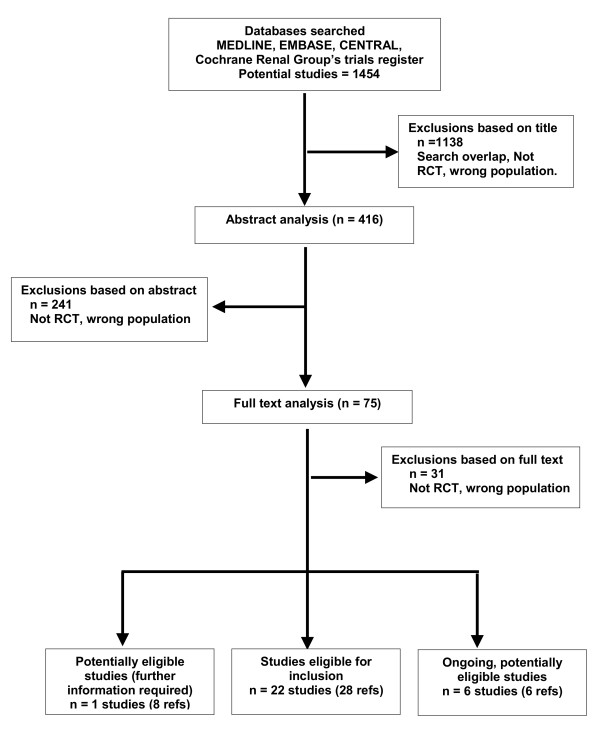
**Flow chart of study selection process**.

Twenty two studies (28 references, 1674 participants) were identified as eligible for inclusion in this review. For remission induction, six studies assess the use of plasma exchange adjunctive therapy [7,29-33], four studies address the use of pulse versus continuous cyclophosphamide treatment [20,34-36] and six further studies consider other potential treatments including rituximab[24,25], mycophenolate[21], lymphocytapheresis[37], immunoadsorption [38] for remission induction or intravenous immunoglobulin for refractory disease[16]. Maintenance treatment is considered by six studies including comparisons of AZA after 3 months of remission induction with continued CPA[39], AZA and MMF[40], AZA and Methotrexate[23], Methotrexate and Leflunomide[22], the use of co-trimoxazole[41] and etanercept[26]. No quasi RCT studies were identified. The demographics of the included patients are included in the review in the Cochrane Library. The numbers of studies identified by the search and subsequently excluded are documented in the flow diagram in Figure 1

### Plasma Exchange

Six studies addressed the adjunctive use of plasma exchange with immunosuppression [29-33]. Inclusion criteria are summarised in Table 1. The treatments tested in each of these studies differed in terms of both the standard immunosuppression and the protocol for plasma exchange (Table 2).

**Table 1:** Inclusion and exclusion criteria for plasma exchange studies

Study Id	Inclusion criteria	Exclusion criteria
Cole 1992	RPGN of undefined aetiology (idiopathic or post infectious disease) with specific pathologic criteria.	Cellular crescents in < 50% non-obsolescent glomeruli. Evidence of serious infection or active ulcer disease.
	Adults (16-75 y), normal sized kidneys SCr > 170 μmol/L and/or increasing by 44 μmoles/l per wk.	
	No evidence of systemic disease or anti-glomerular basement membrane antibody-induced disease.	
	Renal biopsy within 5 days of trial entry	
Jayne 2007	Biopsy-proven ANCA-associated necrotizing GN with acute kidney failure (SCr > 500 μmol/L)	Age <18 or > 80 years.
		Inadequate contraception; pregnancy; previous malignancy; hepatitis B antigenaemia or hepatitis C antibody or HIV infection; other multi-system autoimmune disease; circulating anti-GBM antibody or linear staining of GBM on histology; life-threatening non-renal manifestations of vasculitis.
		Dialysis for > 2 weeks before entry; creatinine >200 uM more than 1 year before entry. > 2 weeks treatment with CPA or AZA; > 500 mg of IV methylprednisolone; plasma exchange within the preceding year; >3 months treatment with oral prednisolone; allergy to study medications.
Glockner 1988	RPGN with >70% crescents on renal biopsy. CrCl < 50 ml/min. Urine output > 200 ml/24 h.	Anti-GBM disease, life threatening conditions, contraindications to immunosuppression, previous treatment with AZA or CPA for >14 days.
Mauri 1985	Histologically proven crescentic GN and rapidly progressive renal impairment.	Less than 60% glomerular involvement, primary glomerulopathies, transplanted kidneys, SLE, HSP.
Pusey 1991	Focal necrotizing GN with crescents (Wegener's granulomatosis, systemic vasculitis, polyarteritis, idiopathic RPGN)	Anti-GBM disease, SLE, Henoch-Schonlein Purpura, chronic GN Previously treated with IV MP, oral CPA or PE
Rifle 1980	New onset RPGN with > 50% glomerular crescents.	Goodpasture's syndrome; IgA nephropathies; SLE; systemic disease.

**Table 2:** Interventions in the plasma exchange studies

Study ID	Treatment	Control	Study outcomes
Cole 1992	PE: at least 10 PE treatments within 16 days of trial entry. One plasma volume with complete replacement using 5% albumin + crystalloid. Immunosuppression: as for control group	Immunosuppression IV MP 10 mg/kg/d for 3 days followed by prednisone 1.4 mg/kg/d for next 4 days and then tapered to 1 mg/kg/d over 2 weeks 0.35 mg/kg/d at 1 month and 0.25 mg/kg/d at 2 months. AZA 1.5-3.0 mg/kg/d with dose adjustment as necessary to ensure neutrophil count of 2.0 × 10^9^/L or greater	1. renal pathology 2. Patients on dialysis at randomisation - dialysis at 1, 3, 6, 12 months 3. Renal function in patients not on dialysis - 1, 3, 6, 12 months 4. Change in SCr 5. Adverse events (serious infections, gastrointestinal bleeding) 6. Mortality
Jayne 2007	Seven PE of 60 ml/kg in first 2 weeks after diagnosis Immunosuppression as for control group	Three pulses of 1000 mg IV MP followed by oral CPA and a tapering regimen of prednisolone.	1. Mortality 2. Dialysis 3. Side effects 4. Serum creatinine at 12 months
Glockner 1988	Nine 50 ml/kg PE over 4 weeks replaced with 3-5% Albumin solution. Immunosuppression as for control group	CPA 3 mg/kg/d plus AZA 1 mg/kg/d for 1 week then AZA 2 mg/kg/d. 6-methyl prednisolone 1.5 mg/kg/d for 14 days reducing in 4 mg/d steps to maintenance 8 mg/d.	1. Mortality at 6 months 2. Dialysis at 6 months 3. SCr at 4 weeks, 8 weeks and 6 months 4. Adverse events including serious infections, GI haemorrhage and anaphylaxis
Mauri 1985	PE alternate days for 6 treatments. Exchanges of at least 3.5 L replaced with 3.5% Albumin and 2 units FFP. Immunosuppression as for control group	CPA 2 mg/kg/d and Prednisolone 1 mg/kg/d. Doses reduced to half after 8 weeks. Prednisolone dose tapered progressively. CPA dose reduced to 0.5 mg/kg/d after 2 months then stopped after month 4.	1. Mortality 2. Dialysis post treatment, at 3 months and 12 months after treatment 3. SCr after treatment and 6 months later
Pusey 1991	PE: 5 × 4 L exchanges of 5% albumin (plasma protein fraction) within first week. Two units of fresh frozen plasma were given at end of exchange. Total number of exchanges determined by clinical response. Immunosuppression as for control group	Induction therapy: 8 weeks of: 1. 60 mg/d prednisolone, reduce to 30 mg/d at week 3, taper slowly. 2. CPA 3 mg/kg/d or 2 mg/kg/d for those over 55 years 3. AZA 1 mg/kg/d or no AZA for those over 55 years Maintenance therapy: CPA stopped after 8 weeks in those with remission and AZA increased to 2-3 mg/kg/d, together with tapering doses of prednisolone"	1. Improvement (fall in SCr > 25% or rise in CrCl > 25%; recovery of renal function in those initially on dialysis) 2. SCr 3. Dialysis 4. Death 5. Adverse event
Rifle 1980	PE. Five sessions during 5 successive days, then 3 sessions/week until 15 days after SCr reached a plateau. Treatment could not exceed 2 months. 1.5 plasma volumes exchanged. Immunosuppression as for control group	IV pulse MP (15 mg/kg/d for 3 days, tapered to 15 mg/d for 3 days, then 3 new pulses, then 15 mg/d for 7 weeks. CPA 2-3 mg/kg/d for 2 months. Calcium heparinate 9 days after renal biopsy for the duration of the study.	1. Dialysis 2, 6 12, 24 months 2. CrCl at 2, 6 and 12 months. 3. Recovery (off dialysis) according to initial SCr level 4. Recovery (off dialysis) according to initial % of crescents 5. Death 6. Circulating immune complexes 7. Pathology changes 8. Adverse events (septicaemia)

**Figure 2:**
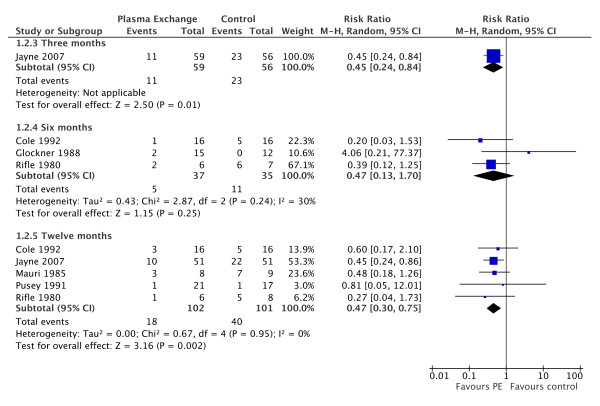
**Forest plot showing the risk of requiring renal replacement therapy at 3, 6 or 12 months after induction treatment for vasculitis in patients treated with and without plasma exchange**. At 3(1 study) and 12 months (5 studies) there is a significantly lower risk of requiring dialysis in patients treated with plasma exchange (PE).

Plasma exchange significantly reduced the need for dialysis at three months (1 study; RR 0.45 95% CI 0.24 to 0.84; P = 0.01; NNT = 5) and 12 months (5 studies; RR 0.47; 95% CI 0.30 to 0.75; P = 0.002; NNT = 5; I^2 ^= 0%) post-treatment (Figure 2). Jayne 2007 included patients with SCr > 500 uM and showed a significant reduction in the need for dialysis at three and 12 months. For all other outcomes (death, SCr and side effects) there was no significant difference between the treatment groups.

### Pulse versus continuous cyclophosphamide

Four studies assessed pulse versus continuous administration of CPA [20,34-36]. Systemic, rather than specifically renal, vasculitis was included. Raw data has been obtained from Adu et al and those patients with polyarteritis nodosa have been excluded from this analysis. Inclusion criteria are summarised in Table 3. The treatment regimens in these studies also differed, as summarised in Table 4.

**Table 3:** Inclusion criteria for the pulse versus continuous CPA studies

Study ID	Inclusion criteria	Exclusion criteria
Adu 1997	Patients 15-70 y with new-onset systemic necrotizing vasculitis. Wegener's granulomatosis, classical polyarteritis nodosa and microscopic polyarteritis diagnosed by histological or radiological evidence.	
De Groot 2009a	newly diagnosed Wegener's Granulomatosis, microscopic polyangiitis, or renal-limited microscopic polyangiitis renal involvement: at least one of: serum creatinine >150 umol/l and <500 μmol/l, biopsy evidence of necrotizing GN, erythrocyte casts, or haematuria and proteinuria, confirmatory histology or ANCA positivity	Other multi-system autoimmune disease; hepatitis B or C virus or HIV infection/Serum creatinine > 500 μmol/l; previous cancer; pregnancy; age under 18 yrs or older than 80 yrs.
Guillevin 1997	Age > 15 years New diagnosis of systemic WG diagnosed clinically based on the presence of multi-organ involvement. Mono-visceral involvement representing a potential risk or severe morbidity of fatality. Histopathologic characterization of necrotizing granulomatosis vasculitis or evidence of either granulomatous inflammation and vasculitis or segmental necrotizing GN	NS
Haubitz 1998	New diagnoses of WG and MPA and renal involvement. Biopsy performed	Age < 18 y, pregnancy, HIV, malignancy, SCr > 200 μmol/L more than 1 year before presentation, cytotoxic drug therapy for > 1 week before start of study, HD for > 10 days before start of study.

**Table 4:** Interventions in the pulse versus continuous CPA studies

Study ID	Treatment	Control	Study outcomes
Adu 1997	CPA 15 mg/kg and MP were given IV at 0, 2 and 4 weeks. The same dose was then given as oral pulses over a 3-day period. The interval between pulses was gradually increased.	initial treatment - 0.85 mg/kg prednisolone then tapering according to a predefined schedule for 72 weeks. CPA 2 mg/kg/d given until a clinical decision that remission had been achieved at which point CPA was stopped and AZA commenced at 1.5 mg/kg/d	1. Complete and partial remission 2. Relapse 3. Adverse events 4. Treatment failure 5. Chronic dialysis
De Groot 2009a	3 iv pulses of CPA 15 mg/kg 2 weeks apart followed by 3 weekly pulses (15 mg/kg iv or 5 mg/kg orally for 3 days) until remission then for another 3 months. Mac dose 1.2 G. Reductions for age > 60 yrs and serum creatinine > 300 uM and for previous low leukocyte nadir.	oral CPA 2 mg/kg/d to remission then 1.5 mg/kg for further 3 months. Max oral dose 200 mg. Reductions for age > 60 yrs and leukopenia Both groups received Azathioprine 2 mg/kg/d orally after induction therapy until month 18. Prednisolone 1 mg/kg orally tapered to 12.5 mg/d at the end of month3 and 5 mg at end of study	1. Time to Remission 2. Change in renal function 3. Adverse events 4. Cumulative dose of CPA
Guillevin 1997	Initial regimen: IV MP 15 mg/kg/d for 3 days. IV CPA 0.7 g/m^2 ^day 4. Oral prednisolone 1 mg/kg/d from day 4. IV pulse CPA: mean dose 0.7 g/m^2 ^adjusted for count and renal function, administered every 3 weeks until complete remission and 1 year thereafter. Then every 4 weeks for 4 months, every 5 weeks for 4 months and every 6 weeks until discontinuation after 2 years if treatment. Adjusted up or down based on neutrophil count.	Initial regimen: IV MP 15 mg/kg/d for 3 days. IV CPA 0.7 g/m^2 ^day 4. Oral prednisolone 1 mg/kg/d from day 4. Oral CPA: 2 mg/kg/d on day 10 after initial CPA pulse, after neutrophil nadir had been reached. 1 year after complete remission, oral CPA was tapered by 25% every 4 months until discontinuation. Dose adjusted up or down based on neutrophil count.	1. Treatment failure 2. Complete remission 3. Partial remission 4. Relapse 5. Death 6. Side effects
Haubitz 1998	Steroid regime: Days 1-3, 0.5 g IV MP. Day 4 1 mg/kg/d oral prednisolone. Tapered from day 15. IV Pulse CPA - 0.75 g/m^2 ^every 4th week. If CrCl < 30 ml/min, initial dosage was 0.5 g/m^2 ^ and increased to 0.75 g/m^2 ^provided leukocyte counts remained > 3000/ml	Steroid regime: Days 1-3, 0.5 g IV MP. Day 4 1 mg/kg/d oral prednisolone. Tapered from day 15. Oral daily CPA - 2 mg/kg/d. If CrCl < 30 ml/min, initial dosage started at 1.5 mg/kg/d and increase to 2 mg/kg/d after 2 weeks provided leukocyte counts were > 3000/ml CPA dose reduced in steps of 0.5 mg/kg, unless leukocyte count was < 2500/ml then dose reduced by 50%, and if less than 1500/ml drug was withheld until increased to 2500/ml	1. Complete remission 2. Partial remission 3. Relapse 4. Serious infection

**Figure 3:**
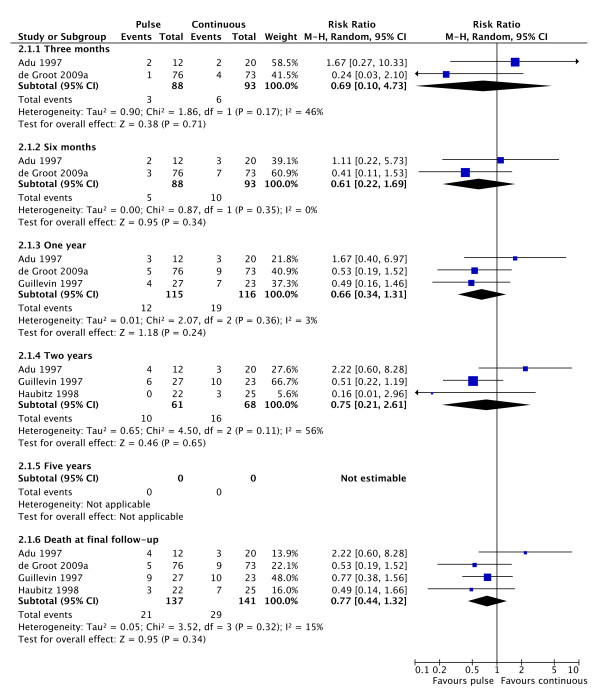
**Forest plot showing the risk of death at 3, 6 and 12 months and 2 and 5 years and at final follow-up in patients treated with pulse or continuous cyclophosphamide**. At no time point is there any significant difference between the treatment groups.

**Figure 4:**
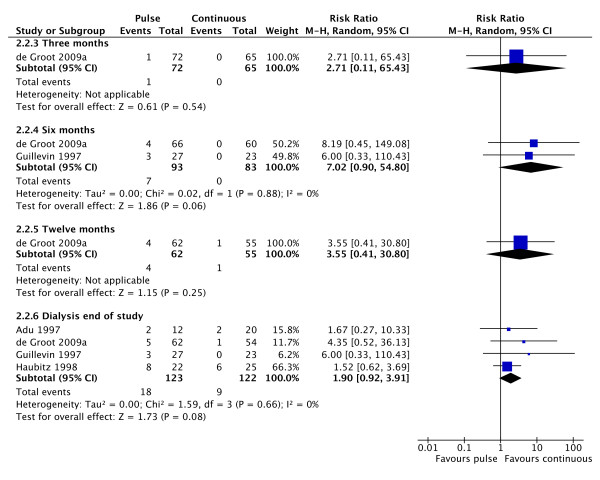
**Forest plot showing the risk ratio of end stage renal failure in patients treated with either pulse or continuous cyclophosphamide therapy at 3, 6 or 12 months and at study end**. Overall more patients require dialysis after treatment with pulse therapy but this does reach statistical significance.

Mortality was not significantly different between the two groups. There was evidence of statistically significant heterogeneity between the studies (Figure 3). There is a trend towards an increase in the number of patients requiring RRT with pulse CPA therapy, with twice as many patients requiring dialysis on this treatment compared to continuous treatment (Figure 4).

**Figure 5:**
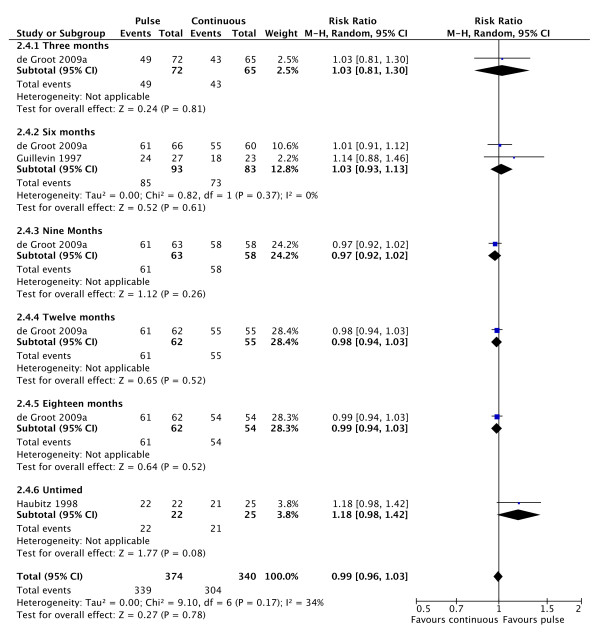
**Forest plot showing the risk ratio of achieving remission in patients treated with pulse or continuous cyclophosphamide at various time points**. There is no change in the rate of remission with pulse cyclophosphamide.

**Figure 6:**
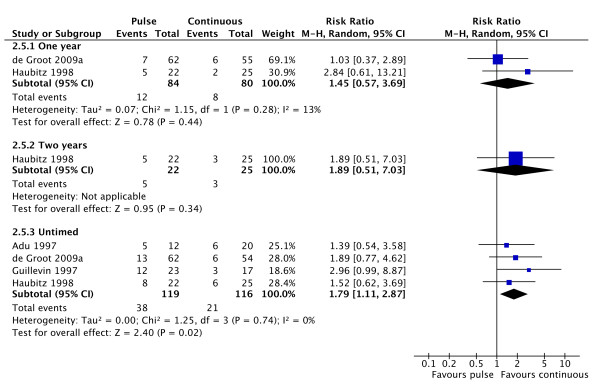
**Forest plot showing the risk ratio of relapse in patients treated with continuous or pulse CPA at 1 and 2 years and at study end**. Overall continuous treatment with CPA results in a reduced rate of relapse.

Remission rates were equivalent for the two interventions (Figure 5; 3 studies; RR 0.99, 95% CI 0.96 to 1.03; P = 0.17; I^2 ^= 34%). There was an increased risk of relapse with pulse versus continuous therapy (Figure 6; 4 studies; RR 1.79, 95% CI 1.11 to 2.87; P = 0.02; NNH = 5; I^2 ^= 0%).

Leukopenia was less common with pulse treatment (4 studies; RR 0.53, 95% CI 0.36 to 0.77; P = 0.0009; NNH = 5; I^2 ^= 0%), and nausea more common (2 studies; RR 2.51, 95% CI 1.07 to 5.89; P = 0.04 NNH = 7; I^2 ^= 0%). Other trends suggested less treatment failure but more serious infections with continuous therapy but these were not statistically significant. There was no significant difference in mortality and SCr.

### Other remission induction studies

Inclusion and exclusion criteria, interventions and primary outcomes for these studies are detailed in tables 5 and 6.

**Table 5:** Inclusion criteria for other remission induction studies

Study ID	Inclusion criteria	Exclusion criteria
Hu 2008	Newly diagnosed active ANCA associated vasculitis. Over 18 years of age with renal involvement with serum creatinine < 500 uM. ANCA positive or ANCA negative with confirmatory renal Biopsy	Cytotoxic drug treatment in 6 months prior. HBV, HCV, HIV or active CMV viral infection, acquired immune deficiency, severe renal failure with creatinine > 500 uM or on renal replacement therapy, life-threatening organ manifestations (lung haemorrhage or CNS involvement), active TB, liver dysfunction, pregnancy or inadequate contraception if female, under age 18 or over 65.
Jones 2008 (RITUXVAS)	A new diagnosis of Wegener's Granulomatosis (WG), Microscopic Polyangiitis (MP) or Renal-Limited Vasculitis (RLV) Renal involvement attributable to active WG, MP or RLV with at least one of the following: Biopsy demonstrating necrotizing glomerulonephritis Red cell casts on urine microscopy or ≥++ haematuria Anti-Neutrophil Cytoplasmic Antibodies (ANCA) positivity; ANCA positivity requires either: Proteinase 3 anti-neutrophil cytoplasmic antibody (PR3-ANCA) by Enzyme-Linked Immunosorbent Assay (ELISA) or a typical anti-neutrophil cytoplasmic antibody (cANCA) pattern by indirect immunofluorescence (IIF), or both Myeloperoxidase-anti-neutrophil cytoplasmic antibody (MPO-ANCA) by ELISA. A positive peri-nuclear anti-neutrophil cytoplasmic antibody (pANCA) by IIF requires confirmation by MPO-ANCA ELISA.	1. Previous cyclophosphamide, (greater than two weeks of an oral or intravenous [IV] pulse cyclophosphamide regimen) 2. Co-existence of another multi-system autoimmune disease, e.g. SLE, Churg Strauss syndrome, Henoch Schonlein purpura, rheumatoid vasculitis, essential mixed cryoglobulinaemia, anti-glomerular basement membrane antibody positivity 3. Hepatitis B antigen positive or hepatitis C antibody positive 4. Known HIV positive (HIV testing will not be a requirement for this trial) 5. Previous malignancy (usually exclude unless agreed with trial co-ordinator) 6. Pregnancy, breast feeding or inadequate contraception if female 7. Allergy to a study medication 8. Live vaccine within last four weeks
Stone 2009 (RAVE)	Weight of at least 88 lbs (40 kg). Diagnosis of Wegener's granulomatosis (WG) or microscopic polyangiitis (MPA) according to the definitions of the Chapel Hill Consensus Conference. Newly diagnosed patient of WG or MPA OR must be experiencing a disease flare characterized by: (a) active disease with a Birmingham Vasculitis Activity Score for Wegener's granulomatosis (BVAS/WG) of 3 or greater that would normally require treatment with CPA; OR (b) disease severe enough to require treatment with CPA; OR (c) must be positive for either PR3-ANCA or MPO-ANCA at the screening. Willing to use acceptable forms of contraception for the duration of the study and for up to 1 year after stopping study medications. Willing to report pregnancies (female participants or male participants' partners) occurring at any time during the study and for up to 1 year after stopping study medications. Parent or guardian willing to provide informed consent, if applicable.	Churg-Strauss Syndrome Limited disease that would not normally be treated with CPA. Mechanical ventilation because of alveolar haemorrhage History of severe allergic reactions to human or chimeric monoclonal antibodies. Active systemic infection. Deep-space infection, such as osteomyelitis, septic arthritis, or pneumonia complicated by pleural cavity or lung abscess, within 6 months prior to study entry. History of or current hepatitis B or C infection. HIV infected. Acute or chronic liver disease. History of or active cancer diagnosed within the last 5 years. History of anti-glomerular basement membrane (anti-GBM) disease. Other uncontrolled disease, including drug and alcohol abuse. Pregnancy or breastfeeding.
Jayne 2000	Prior diagnosis of Wegener's granulomatosis or microscopic polyangiitis ANCA positivity at diagnosis. Active vasculitis with a requirement for further therapy. At least 2 months treatment with prednisolone and cyclophosphamide or azathioprine Age 18 or over	IVIg in previous 3 months history of anaphylaxis to matched blood products selective IgA deficiency RPGN (20% rise in creatinine in 2 weeks) or pulmonary haemorrhage.
Furuta 1998	Biopsy proven rapidly progressive GN.	None Stated
Stegmayr 1999	RPGN ≥ 50% crescents; included WG, Goodpasture's syndrome, MPA	HIV, hepatitis A, B or C virus, severe congestive heart failure, malignancy, septicaemia.

**Table 6:** Interventions in other remission induction studies

Study ID	Treatment	Control	Study outcomes
Hu 2008	MMF 2 G/d (1.5 G if weight < 50 kg) for 6 months Immunosuppression as for control group	Intravenous CPA for 6 months as 0.75-1.0 G/m^2^, modified depending on WCC nadir Iv MethylPrednisolone 0.5 G daily for three days both groups followed by oral prednisolone at 0.6-0.8 mg/kg/d for 4 weeks tapered by 5 mg/wk to 10 mg/d	1. Remission rate at 6 months. Defined as no clinical signs of vasculitis, improved or stable renal function, no active urinary sediment and BVAS score 0. 2. Changes in renal function, side effects
Jones 2008 (RITUXVAS)	Rituximab, 375 mg/m^2 ^IV once a week for 4 weeks (i.e. 4 doses total), with 2 doses of cyclophosphamide 15 mg/kg, 2 weeks apart given with the 1^st ^and 3^rd ^rituximab dose. All patients received 1 g IV methylprednisolone, then same daily oral corticosteroid regimen.	CPA 15 mg/kg for 3-6 months (6-10 doses total) IV for remission induction. AZA for remission maintenance. All patients received 1 g IV methylprednisolone, then same daily oral corticosteroid regimen.	1. Primary i) Sustained remission (BVAS = 0 at 6 months and sustained for 6 months). ii) Severe adverse events at 2 years. 2. Secondary a. Efficacy Response rate at 6 weeks (BVAS < 50% baseline) Remission at 6 months (BVAS = 0 for 2 months by 6 months) Time to remission (BVAS = 0) Relapses (all relapses and major/minor) BVAS area under the curve Change in GFR Change in SF-36 Change in VDI b. Safety Severe adverse events at 6 weeks and 6 months. All adverse events. Death. Prednisolone cumulative dose. CPA cumulative dose 3. Tertiary. Human anti-chimeric antibody testing Correlation of B cells with disease activity Change in ANCA and disease activity Histopathology predictors of outcome.
Stone 2009 (RAVE)	rituximab (375 mg/m^2^) infusions once weekly for 4 weeks and cyclophosphamide (CPA) placebo daily for 3 to 6 months. Remission Maintenance: discontinue CPA placebo and start oral azathioprine (AZA) placebo daily until Month 18 Both groups: Iv MP 1-3 G then Prednisolone 1 mg/kg/d reduced to 40 mg month 1, tapered to discontinue at the end of month 6	rituximab placebo infusions once weekly for 4 weeks and CPA daily for 3 to 6 months Remission Maintenance: discontinue CPA and start AZA daily until Month 18. Both groups: Iv MP 1-3 G then Prednisolone 1 mg/kg/d reduced to 40 mg month 1, tapered to discontinue at the end of month 6	Primary outcome measures Complete remission during the first 6 months after randomisation Secondary outcome measures Adverse events
Jayne 2000	IVIg 0.4 G/kg/d for 5 days	Placebo (identical injections)	Primary outcome: treatment response. BVAS reduction of 50% between entry and 3 months Secondary outcomes: fall in BVAS, CRP and ANCA, relapse frequency between 3 and 12 months, reduction in immunosuppressive drug doses and adverse effect
Furuta 1998	Three 1 hour sessions of lymphocytapheresis on alternate days in each of three consecutive weeks. Immunosuppression as for control group	1 G MP iv for 3 consecutive days in each of three consecutive weeks. BOTH GROUPS: Prednisolone 20 mg/day and CPA 50 mg/day.	1. SCr 4 weeks post treatment 2. Mortality
Stegmayr 1999	Immunoadsorption: At least 2 plasma volumes were removed. Median of 6 sessions. Immunosuppression as for control group	3 PE in first 5 days of at least 1 plasma volume. 4% Albumin as replacement. Median of six sessions. All patients received immunosuppression with pulse MP and oral or iv CPA 2 mg/kg/day. CPA continued for 8 weeks or longer if ANCA positive.	1. CrCl and SCr 2. Responder (CrCl improved by at least 20 ml/min or patient could leave dialysis) 3. Adverse events 4. Dialysis 5. Death

### Rituximab vs cyclophosphamide alone for remission induction

Two studies have been published in abstract form, the RITUXVAS and RAVE studies[25,24]. In RITUXVAS, 44 patients with newly diagnosed AAV with renal involvement were randomised 3:1 to receive rituximab or CPA. Four doses of Rituximab were given with 2 doses of CPA in the rituximab arm of the study. Primary end point was sustained remission at 6 months. There was no difference demonstrated for any outcome including remission and adverse events.

**Figure 7:**
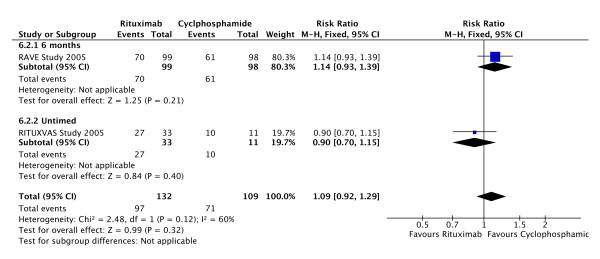
**Forest plot showing the risk ratio for induction of remission in patients treated with or without rituximab at 6 months and at study end**. There is no significant difference between the groups.

The RAVE study distinguished itself by being a randomised blinded study involving the use of placebo rituximab infusions and CPA and AZA tablets. One hundred and ninety seven patients with severe AAV were recruited. Only 99 of these patients were classed as having major renal involvement. Six month results are reported in abstract form only. Primary outcome was disease remission in the absence of prednisolone therapy at 6 months. The results reported here are disease remission at 6 months on Prednisolone 10 mg/d or less. As shown in Figure 7 remission rates were similar between the two groups.

### Mycophenolate Mofetil for remission induction

The use of MMF for remission induction is compared with CPA by Hu et al [21]. Thirty five patients with newly diagnosed ANCA associated vasculitis and moderate renal involvement were randomised to either 2 g/d MMF or standard intravenous CPA monthly pulses for 6 months.

The primary outcome measure was remission rate at 6 months. MMF was found to be equivalent to CPA in this limited population for remission at 6 months (1 study; risk ratio 1.65; 95% confidence interval 0.94 to 2.90). There were no data on relapse rate on either treatment. Side effects appeared similar on both agents (risk ratio 0.65; 95% confidence interval 0.20 to 2.14)

### Intravenous immunoglobulin for treatment of persistent disease

IVIg treatment for persistent disease has been studied by a single RCT [16]. Thirty four patients with persistent disease activity were randomised to IVIg 0.4 g/kg/d for 3 days or to identical placebo injections. The primary outcome was a response to treatment defined as a 50% reduction in BVAS (Birmingham Vasculitis Activity Score).

The use of IVIg demonstrated a therapeutic response in more patients at 3 months when compared with placebo. (1 study; risk ratio 2.33; 95% confidence interval 1.18 to 4.61). Benefit was not demonstrated beyond 3 months. There was no demonstrated difference in mortality or rate of relapse.

### Immunoadsorption and Lymphocytapheresis

Immunoadsorption was compared to plasma exchange in a single study [38]. 44 patients were randomised to receive either immunoadsorption or plasma exchange. A median of six sessions of each treatment were delivered. Immunosuppression was otherwise the same in the groups with standard CPA and prednisolone treatment. Primary outcome was renal function and response to treatment.

There was no detected difference in efficacy between immunoadsorption and plasma exchange, as assessed by mortality, the need for dialysis and SCr.

Lymphocytapheresis is compared to 3 days of iv methylprednisolone as adjunctive therapy by Furuta et al [37]. Twenty four patients were randomised with the primary outcome being serum creatinine 4 weeks post treatment.

The use of lymphocytapheresis showed a highly significant reduction in SCr at four weeks (1 study; MD -2.10, 95% CI -2.64 to -1.56). Other outcomes showed a tendency to lower mortality (1 study; RR 0.40, 95% CI 0.10 to 1.67) and fewer patients on dialysis (1 study; RR 0.33, 95% CI 0.04 to 2.77). However, the confidence intervals for these are wide and clinical significance uncertain. No further data has been published to support this study.

### Maintenance Studies

Inclusion and exclusion criteria are detailed in Table 7 and the interventions in Table 8.

**Table 7:** Inclusion Criteria for studies of maintenance treatment

Study ID	Inclusion criteria	Exclusion criteria
Jayne 2003	Diagnosis of WG, MPA or renal limited vasculitis. Renal involvement, other threatened loss of function of vital organ, or both. ANCA positivity. ANCA negative patients enrolled with biopsy evidence of vasculitis.	Cytotoxic drug in previous year. Other multi-system autoimmune disease, hepatitis B antigenaemia, hepatitis C, HIV infection, cancer, pregnancy. Age: <18 or > 75 years SCr >500 μmol/L
Stegeman 1996	RPGN on biopsy plus lung disease compatible with WG. Disease limited to respiratory tract. Meeting Chapel Hill consensus but not criteria 1	Previous reactions to co-trimoxazole. GFR < 30 ml/min Long term treatment with antibiotics
Metzler 2007	18 to 75 years of age. Diagnosis of generalized WG Successful induction therapy with prednisolone and CPA.	Bone marrow insufficiency (leukopenia < 4000/μl, Hb < 10 g/dl, thrombocytopaenia > 100,000/μl) serum creatinine > 1.3 mg/dl (115 uM), malignancies, hepatitis B or C or HIV positivity, pregnancy or breast feeding, inadequate contraception, chronic liver disease or alcohol abuse, active gastric ulcer, lack of compliance, further coexisting autoimmune diseases or treatments interfering with the MTX/LEF medication.
WGET 2005	Patients with BVAS/WG score of 3 or more. Stratified to severe (life-threatening manifestations including RPGN, alveolar haemorrhage or neuropathy) or limited (skin, joints, sinus or mild renal abnormalities). At least 2 of 5 modified criteria of the American College of Rheumatology for classification of Wegener's Granulomatosis. Either new or established disease	None stated
Pagnoux 2008	Patients over 18 years old. Newly diagnosed Wegener's Granulomatosis and Microscopic Polyangiitis. Successful remission induction.	use of steroids for more than 1 months prior to CPA therapy, co-existence of another systemic disease, cancer (unless in remission for more than 3 years), HIV, Hep B or C virus infection, contraindication to study drugs, pregnancy, absence of contraception in pre-menopausal women, mental or physical disabilities abrogating ability to consent. Patients not entering remission were not randomised.
Hiemstra 2009 (IMPROVE)	Newly diagnosed patients with WG, MPA or renal-limited vasculitis. ANCA positivity. Age 18 to 75 years	Any cytotoxic drug within previous year. Co-existence of another systemic autoimmune disease, e.g. SLE. Hepatitis B or Hepatitis C infection HIV positivity. Failure to achieve remission after 6 months of CPA therapy. Failure to control progressive disease with induction protocol. Malignancy. Pregnancy or inadequate contraception. End stage renal failure unless active extra-renal disease requires treatment Inability for informed consent

**Table 8:** Interventions for studies of maintenance treatment

Study ID	Treatment	Control	Study Outcomes
Hiemstra 2009 (IMPROVE)	Mycophenolate mofetil 2 G/d for 42 months	azathioprine 2 mg/kg/d for 42 months	1. Time to first relapse 2. relapse rate 3. Rate of side-effects and intolerance 4. cumulative doses (AZA, CS, MMF), 5. AUC for BVAS, SF-36 or VDI 6. Evolution of titres of ANCA and CRP
Pagnoux 2008	All patients received identical remission induction therapy. Pulse MP 15 mg/kg for 3 days. Oral prednisolone 1 mg/kg/d for 3 weeks, tapered to 12.5 mg at 6 months. Pulse CPA 0.6 G/m^2^, 3 doses at 2 week intervals then every 3 weeks until remission, 3 further consolidation doses at 3 week intervals. Azathioprine 2 mg/kg/d Maintenance therapy continued for 12 months then withdrawn over 3 months. Trimethoprim-sulfamethoxazole 320/1600 daily recommended for WG patients for additional 2 years.	Methotrexate 0.3 mg/kg/week, increasing every week by 2.5 mg to 25 mg/week. Folinic Acid 25 mg or Folic Acid 5 mg given 48 hours after Methotrexate. Maintenance therapy continued for 12 months then withdrawn over 3 months. Trimethoprim-sulfamethoxazole 1600/320 daily recommended for WG patients for additional 2 years.	Primary end point: 1. adverse reaction causing death or leading to discontinuation of the study drug. Secondary end points: 2. any adverse event, 3. severe adverse event, 4. relapse, 5. relapse free survival, 6. event free survival, 7. quality of life.
WGET 2005	Etanercept 25 mg twice weekly by subcutaneous injection Immunosuppression as for control group.	Twice weekly placebo injection Severe disease: Cyclophosphamide 2 mg/kg/d. Replaced with methotrexate if in remission at 3 to 6 months. Limited disease: Methotrexate 0.25 mg/kg/week to maximum of 25 mg/week. 12 months after remission, MTX dose cut by 2.5 mg each month. Prednisolone was given to patients with severe and limited disease starting at 0.5 to 1.0 mg/kg/d. Tapered to 0 mg at 6 months if no relapse. Patients in remission with creatinine > 2 mg/dl received Azathioprine 2 mg/kg/d, decreased after 12 months in remission by 25 mg each month.	1. Sustained remission. BVAS/WG score 0 for at least 6 months. 2. Number and rate of flares during treatment, percentage of patients with sustained low level of disease activity (BVAS/WG < 3 for at least 6 months), percentage of patients with a remission, cumulative area under the curve for the BVAS/WG, adverse events, quality of life.
Metzler 2007	Leflunomide. Loading dose of 100 mg/d for 3 days, followed by 20 mg/d from day 4 to end of week 4. Then increased to 30 mg/d thereafter. Prednisolone as per methotrexate group.	Methotrexate: 7.5 mg/week weeks 1 to 4. 15 mg/week for weeks 5 to 8. 20 mg/week after week 8. Prednisolone allowed at a dose of 10 mg/d or less. In the absence of disease activity, the dose was tapered by 2.5 mg/month to 5 mg, then by 1 mg/month.	1. Number of major and minor relapses. 2. Disease Extent Index (DEI), BVAS, patient self assessment of quality of life (SF-36), cANCA titre, ESR, CRP.
Stegeman 1996	Co-trimoxazole 960 mg twice daily	Placebo	1. Death 2. Remission
Jayne 2003	CPA 1.5 mg/kg/d from remission. Switched to AZA 1.5 mg/kg/d 12 months after study entry. Immunosuppression as for control group.	After remission induction, AZA 2 mg/kg/d with Prednisolone 10 mg/d. BOTH GROUPS: Remission induction with oral CPA 2 mg/kg/d and prednisolone 1 mg/kg/d tapered to 0.25 mg/kg/d by 12 weeks. From 12 months both groups received AZA 1.5 mg/kg/d and prednisolone 7.5 mg/d.	1. Relapse by 18 months 2. Side effects including leucopenia and infections

### Azathioprine as maintenance therapy

The use of AZA to reduce exposure to CPA is assessed by Jayne et al [39]. Patients were treated with standard induction therapy of CPA 2 mg/kg/d and prednisolone 1 mg/kg/d. At remission, 155 patients were randomised to either AZA 2 mg/kg/d and prednisolone 10 mg/d or continued CPA 1.5 mg/kg/d until 12 months, after which all patients were treated with AZA 1.5 mg/kg/d. Primary outcomes were relapse by 18 months and side effects of treatment.

The introduction of AZA after remission did not alter the rate of relapse at 18 months compared to the group who remained on CPA (1 study; RR 1.13, 95% CI 0.51-2.5). Leukopenia was significantly less likely on AZA (1 study; RR 0.65, 95% CI 0.42 to 0.99) though this was not reflected in an increase in infective complications in patients on CPA (1 study; RR 1.03, 95% CI 0.51 to 2.06).

### MMF vs Azathioprine for maintenance treatment

One hundred and seventy four patients with newly diagnosed AAV were randomised after successful remission induction to receive either MMF or AZA[40]. Primary outcome was time to first relapse. Time to relapse was shorter for the MMF group but this has not yet been reported in detail. The abstract published states the hazard ratio of relapse in the MMF group was 1.7 (95% CI 1.09-2.85; p = 0.03) suggesting that MMF treatment results in a higher relapse rate in comparison with AZA.

### Azathioprine vs Methotrexate for maintenance treatment

Pagnoux et al compared AZA and MTX for maintenance treatment[23]. One hundred and twenty six patients treated to remission with standard doses of intravenous CPA and steroids were randomised to either AZA 2 mg/kg/d or MTX 0.3 mg/kg/wk increasing by 2.5 mg/week to a total of 25 mg/wk. Primary outcome was adverse reaction causing death or leading to discontinuation of the study drug. Secondary outcomes were any adverse event, severe adverse event, relapse, relapse free survival, event free survival and quality of life.

There was no difference between the treatments for primary outcome (1 study; RR = 0.58 (95% CI 0.25-1.38). Both treatments gave similar results for all the secondary outcomes.

### Leflunomide vs Methotrexate for maintenance treatment

Leflunomide was compared with methotrexate for remission maintenance [22]. Fifty four patients successfully treated to remission with prednisolone and CPA, were randomised to either leflunomide or methotrexate treatment. The primary outcome was the number of relapses after 24 months on treatment.

Leflunomide was shown to be superior to methotrexate for relapse prevention in a time to event analysis in Metzler 2007. Our analysis suggests Leflunomide may be superior but does not reach statistical significance (1 study; risk ratio 0.50; 95% confidence interval 0.22 to 1.11). Major relapses were also reduced (risk ratio 0.15; 95% confidence interval 0.02 to 1.17) and side effects similar on both treatments (risk ratio 2.71; 95% confidence interval 0.49 to 14.85). There were multiple methodological difficulties with this study addressed in the discussion section.

### Etanercept as adjunctive therapy

The use of etanercept as adjunctive therapy in AAV was investigated by the Wegener's Granulomatosis Etanercept Trial Research Group [26]. One hundred and eighty patients with new or established WG were randomised to receive either etanercept 25 mg twice weekly by subcutaneous injection or placebo treatment. Immunosuppression with prednisolone and CPA for generalized or methotrexate for limited disease was also given to both groups. Primary outcome was sustained remission with a BVAS/WG of 0 for at least 6 months.

Etanercept was not shown to be effective in remission induction or relapse prevention. There was no difference in the induction of sustained remission (1 study; risk ratio 0.93; 95% confidence interval 0.77 to 1.11) or relapse rate (1 study; risk ratio 0.93; 95% confidence interval 0.56 to 1.56). Moreover, the incidence of solid cancers detected during the study was higher in the etanercept arm.

### Co-trimoxazole for relapse prevention

Stegeman [41] studied the efficacy of co-trimoxazole in preventing relapse of patients with ANCA associated vasculitis. Eighty patients with vasculitis and either RPGN on biopsy or otherwise meeting the Chapel Hill Consensus were randomised to co-trimoxazole 960 mg twice daily or placebo. Primary outcomes were death, remission and relapse rate.

**Figure 8:**
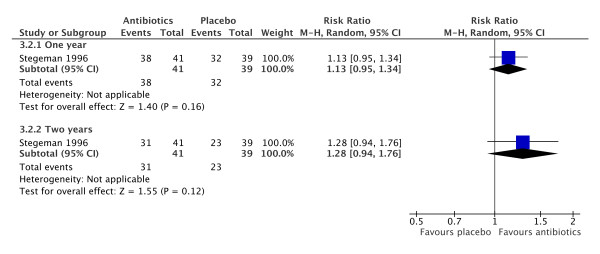
**Forest plot of risk ratio for relapse in patients treated with or without co-trimoxazole at 1 and 2 years**. There is no statistically significant difference between the groups.

The reduction in the number of relapses in patients treated with co-trimoxazole was a significant result on the basis of life-table analysis, giving a reported RR of relapse of 0.40 (95% CI 0.17 to 0.98). Our review, using a fixed time analysis, suggests a smaller difference between the groups with a higher probability of maintaining a remission on antibiotics (1 study; RR 1.28, 95% CI 0.94 to 1.76) although this was not statistically significant (Figure 8).

### Risk of bias in included studies

A summary of quality measures is shown in Table 9. Most of the studies in this review were not blinded. Only three reported blinding of patient, physicians and outcome assessors. The majority of reports do not contain adequate data to clearly indicate the risk of bias. Intention to treat analysis was performed in 10 of 18 studies and follow-up was good, ranging from 82 to 100%.

**Table 9:** Summary of quality measures of included studies

Study ID	Randomisation method	Allocation concealment	Blinding: Participants	Blinding: Investigators	Blinding: Outcome assessors	Blinding: Data assessors	ITT	% Follow-up
Adu 1997	Computer generated random numbers. Stratified for renal function	A	No	No	No	No	Yes	100
Cole 1992	computer-generated random numbers after informed consent Stratified by urine volume (< 400 ml/24 h), need for dialysis and > 50% glomeruli sclerosed	B	No	No	Yes	No	No	97
De Groot 2009a	computer generated, performed centrally by permuted blocks of 4, stratified by country and disease. assigned randomly 1:1 to treatments.	A	No	No	No	Yes	Yes	83
Furuta 1998	NS	B	No	No	NS	NS	Yes	100
Jayne 2007	NS	B	No	No	NS	NS	Yes	100
Glockner 1988	By telephone with statistician	B	No	No	NS	NS	No	84
Guillevin 1997	NS	B	No	No	NS	NS	No	93
Haubitz 1998	Stratified at diagnosis	B	No	No	NS	NS	No	84
Jayne 2007	Centrally, block design. Stratified by diagnosis	A	NS	NS	NS	Yes	Yes	100
Mauri 1985	NS	B	No	No	NS	NS	Yes	100
Pusey 1991	Stratified for severity according to renal function by random numbers	B	No	No	NS	NS	No	92
Rifle 1980	NS	B	No	No	NS	NS	No	82
Stegeman 1996	Stratified by group and then randomized	B	Yes	Yes	Yes	NS	No	99
Stegmayr 1999	NS	B	No	No	NS	NS	No	83
Hu 2008	Not stated	B	No	No	No	NS	Yes	89
Jayne 2000	Central	A	Yes	Yes	Yes	NS	Yes	100
Metzler 2007	Central	A	No	No	No	No	Yes	96
WGET 2000	Stratified by disease severity and by centre	A	Yes	Yes	Yes	NS	Yes	97
Pagnoux 2008	Permuted blocks of six	A	No	No	No	NS	Yes	100
Hiemstra 2009	NS	NS	No	No	NS	NS	Yes	NS
RAVE	NS	NS	Yes	Yes	NS	NS	NS	NS
RITUXVAS	Central randomisation	Yes	No	No	NS	NS	Yes	100

Allocation concealment: A - adequate. B - Unclear. NS Not Stated.

## Discussion

### Remission Induction

#### Plasma exchange as adjunctive therapy

This meta-analysis shows that plasma exchange confers a significant benefit to many patients with RPGN by reducing the risk of end stage renal failure at 12 months from diagnosis. The RR of 0.47 suggests that the number of patients requiring dialysis may be halved by this intervention. Previous studies have shown an effect in the most severely ill patients. A subgroup analysis in Pusey 1991 showed a benefit for patients requiring dialysis at presentation. More recently, Jayne 2007 has shown a benefit for patients with SCr greater than 500 uM with ANCA associated vasculitis. The majority of patients included in these studies would meet the criteria for having severe AKF (SCr > 500 uM or dialysis required at presentation). It is therefore not clear whether plasma exchange has any impact in patients whose kidney failure is not severe. There was little statistical heterogeneity in all outcomes of these studies with the single exception of SCr at 12 months.

#### Pulse versus continuous CPA

This analysis strongly suggests that, although pulse treatment with cyclophosphamide is equivalent to continuous treatment for remission induction, it results in a higher rate of relapse subsequently. None of the studies in this area have been adequately powered to answer the question of relapse rate since this would require either much larger studies or significantly longer follow-up. We are therefore reliant on the results of meta-analysis to attempt to provide an answer. This answer is less than perfect since it is a meta-analysis of results at different times post treatment across studies with significantly different protocols. In spite of that, there is no evidence of heterogeneity in the outcome, suggesting that the final result is likely to be valid. Though the rates of relapse with pulse CPA treatment are perhaps discouraging, this does not invalidate this mode of treatment. Pulse therapy still delivers a significantly lower total dose of CPA. For those patients who remain in remission, they have clearly benefited in terms of risk of long term side effects.

There is a trend towards more patients requiring dialysis with the use of pulse CPA therapy. This is currently not statistically significant, but the fact remains that there were twice as many patients requiring dialysis after pulse therapy and that this effect is present in all studies. Further studies are required to clarify this question.

CPA treatment was given for three months, approximately six months, one and two years in the four relevant studies. This difference may account for the significant level of statistical heterogeneity detected in mortality and the incidence of serious infections. Pulse therapy also causes significantly more nausea but less leukopenia and serious infections. In the light of data from Jayne 2003, it would seem reasonable to suggest that continuous oral CPA should be limited to three months treatment if the patient has achieved a sustained remission with a change to AZA for maintenance therapy. The optimal regimen for CPA administration for remission induction in ANCA associated vasculitis remains unclear.

#### Rituximab vs cyclophosphamide alone for remission induction

The RITUXVAS and RAVE studies are two well designed studies showing that Rituximab is equivalent to CPA therapy for remission induction whilst side effects occur at a similar frequency albeit possibly in a smaller number of patients with rituximab. Given recent papers suggesting that the side effects of cyclophosphamide are more common than previously thought[42], the threshold for rituximab use is likely to fall, in spite of its expense.

#### Mycophenolate for remission induction

The data currently available on this question remain sparse. The study by Hu et al is encouraging in that it does not show a reduction in remission induction with MMF. The next question is the subsequent relapse rate after MMF use for induction and this has not so far been addressed. If the relapse rate is particularly high, MMF may simply turn out to be an expensive prelude to CPA. The population in this trial is significantly different from those in other studies, most obviously in the proportion of patients with MPO-ANCA and microscopic polyangiitis at 87%. This is significantly different from that reported from Europe where the majority of patients are PR3-ANCA positive. The remission rate is also lower than that achieved in similar studies from Europe with only 44% of patients achieving remission as opposed to over 90% (Jayne 2003). The external validity of the trial and wider applicability of its results remain to be established.

#### Intravenous immunoglobulin use for refractory vasculitis

The single RCT in this area suggests a short term benefit lasting up to 3 months. The treatment can be viewed as a therapy available to help induce remission but has little bearing on the longer term problem of remission maintenance.

#### Lymphocytapheresis and immunoadsorption

This novel treatment described by Furuta 1998 gives some benefit when compared with three weeks of intravenous pulse methylprednisolone with a significantly lower SCr in treated patients. Considering the lack of a comparison with plasma exchange and the recent data suggesting the use of plasma exchange is superior to pulse methylprednisolone, there is currently no compelling reason to consider using this therapy. Immunoadsorption, similarly, appears to have no benefit over the use of plasma exchange.

### Maintenance Therapy

#### Azathioprine vs CPA as maintenance therapy

The use of AZA as maintenance therapy after an initial three month treatment with CPA is strongly supported by the data from Jayne 2003. The number of relapses on AZA is similar to CPA with fewer episodes of leukopenia and similar numbers of infections. As well as the data on reduced leukopenia, the reduction in total dose of CPA is presumed to reduce longer term side effects from CPA such as infertility and neoplasia.

#### Leflunomide or Methotrexate for maintenance therapy

The single study of Leflunomide suggests that this may be an appropriate treatment for patients who are intolerant of Azathioprine. There are problems with interpretation and extrapolation from this study. The dose of methotrexate was increased very slowly. Many commentators felt this to be an inadequate dose, potentially causing the higher relapse rate and reflecting poorly the potential of methotrexate in this area. There were also a high number of adverse events in the leflunomide arm. The study does however give some data on the use of Leflunomide and grounds for its clinical use. Final conclusions are difficult to draw. Further study of leflunomide is warranted as induction therapy and in comparison to Azathioprine as maintenance therapy.

#### Azathioprine vs Methotrexate for maintenance therapy

This study showed that the safety and efficacy profiles of Methotrexate and AZA are comparable. This data clearly places methotrexate as a better maintenance agent that MMF since it gave similar relapse rates to AZA. The dosing regimen for MTX in this study was superior to that in the Leflunomide/Methotrexate study since the rate of rise in dose was faster and the final dose higher.

#### Antibiotics for maintenance of remission

The use of co-trimoxazole to maintain remission was examined by Stegeman 1996. This showed a benefit in reducing the risk of relapse but not on other outcomes. Analysis in the paper by life table analysis showed this result to be statistically significant. On our analysis using fixed time analysis, it is not statistically significant (P = 0.12). Relapses detected in the study were mainly respiratory in nature but 11/23 patients with a relapse also had progressive glomerulonephritis.

#### Etanercept use

The stated aim of the single study into the use of etanercept in systemic vasculitis was to demonstrate that the relapse rate would be reduced. The study failed to show this and also suggested an increase in the incidence of malignancy in treated patients. There is currently no RCT data on the use of infliximab or other anti-TNF agents. There is some possibility that alternative agents may produce significantly different outcomes since their mechanism of action is distinct from that of etanercept. At this point in time there is no randomised controlled trial data supporting their use.

#### Comparison with other reviews

Two previous reviews have covered some of the subjects addressed in this review. Bosch 2007 provides a broad review of the treatment of ANCA associated vasculitis [43]. This includes patients with localized disease and those without renal vasculitis. They include a large number of uncontrolled studies. There was no attempt at meta-analysis. In the area of severe vasculitis with kidney involvement, there is a brief summary of the randomised trial data as included in this review. Their conclusions are similar to ours. de Groot 2001 is a review of the data relating to the use of pulse or continuous CPA for induction of remission of ANCA-associated vasculitis and includes a meta-analysis of the randomised controlled trial data [13]. As such, it performs a similar metaanalysis to ours in this area. There are, however, a number of differences. de Groot 2001 has utilised all the data from the Adu study. We have extracted the data only for patients without polyarteritis nodosa and with more than 0% glomerular involvement. This accounts for some of the differences but not all. de Groot 2001 reports that treatment failure is more likely with continuous treatment with CPA. There are also some differences in results for relapse rate. Our results show that continuous treatment is significantly better at preventing relapse. We have studied relapse as related to the initial number of patients whereas de Groot 2001 have recorded relapses as related to achieved remissions. With the higher treatment failure figures in the continuous arm, de Groot 2001 does not show a significant difference in the overall relapse rate.

As stated in the introduction, a version of this review has previously been published in the Cochrane Library[19]. This is an updated version containing the latest RCT data in several areas not previously published as part of this review. Most significant is the new data on pulse versus continuous cyclophosphamide discussed above. Also included here for the first time are the data on MMF, leflunomide, IVIG, rituximab and etanercept.

## Authors' Conclusions

### Implications for practice

Plasma exchange is effective in patients with severe ARF secondary to vasculitis. On current data, the use of pulse CPA results in an increased risk of relapse when compared to continuous use but a reduced total CPA dose. The use of co-trimoxazole is not supported for prevention of relapse of vasculitis. AZA and MTX are effective as maintenance therapy once remission has been achieved. Whilst there is evidence for the use of MMF as an induction agent, the wider applicability of the single study is questionable. The use of MMF in remission maintenance should be third line after failure of other agents such as AZA and MTX. Etanercept is not recommended for use in vasculitis. Leflunomide may be useful as maintenance therapy but requires further evaluation. IVIG is useful but only as a short term measure.

### Implications for research

Further research is required to address the use of plasma exchange in patients with SCr < 500 uM at presentation. This investigation is currently under way as part of the PEXIVAS study run by the European Vasculitis Study Group. A cost effectiveness analysis is also required for the use of plasma exchange when compared with methylprednisolone. Clearly there is a large difference between the two therapies in terms of cost, ease of use and complications of therapy. As the population of patients evolves over time and patients with milder disease are treated, it is highly likely that any cost effectiveness analysis would suggest that a minor benefit potentially conferred by plasma exchange would come at too great a cost when compared with IV methylprednisolone. The need for dialysis after remission induction treatment with cyclophosphamide requires some clarification. Current studies suggest that pulse CPA may leave more patients on dialysis unnecessarily.

The use of MMF for remission induction is being examined in the current MYCYC study by the European Vasculitis Study group. This is a randomised study comparing oral MMF with intravenous cyclophosphamide. This will give further insight into this area with a population of patients which is likely to have a higher proportion of PR3 ANCA positive patients.

A cost effectiveness analysis is also likely to be required for rituximab. Initial results suggest that there is no major benefit in terms of efficacy with rituximab compared with cyclophosphamide. Cost savings may be evident in avoidance of adverse events but this requires further study.

## Abbreviations

AAV: ANCA associated vasculitis; AKF: acute kidney failure; ANCA: Anti-neutrophil cytoplasmic antibody; ARF: acute renal failure; AZA: Azathioprine; BVAS: Birmingham Vasculitis Activity Score; CI: Confidence interval; CPA: cyclophosphamide; GBM: glomerular basement membrane; IVIg: Intravenous Immunoglobulin; MD: mean difference; MMF: Mycophenolate Mofetil; MPO: Myeloperoxidase; MTX: Methotrexate; NNH: number needed to harm; NNT: number needed to treat; PR3: Proteinase 3; RCT: randomised controlled trial; RPGN: rapidly progressive glomerulonephritis; RR: relative risk; RRT: renal replacement therapy; SCr: Serum Creatinine; SMD: standardised mean difference; TNF: Tumour necrosis factor; WG: Wegener's Granulomatosis.

## Competing interests

The authors declare that they have no competing interests.

## Authors' contributions

GW was involved in the design of the study, conducted the literature searches, obtained and analysed studies and wrote the draft and final versions of the paper. NW was involved in the design of the study conducted the literature searches, obtained and analysed studies and edited the draft and final versions of the paper. JC was involved in the design of the study, adjudication on disagreements in data analysis and editing of draft and final versions of the paper. All authors have read and approved the final manuscript.

## Appendix

### Search methods for identification of studies

Relevant studies were obtained form the following sources

1. The Cochrane Renal Group's specialised register and the Cochrane Central Register of Controlled Trials (CENTRAL) in *The Cochrane Library*, (Issue 1, 2008). CENTRAL and the Renal Groups specialised register contain the hand searched results of conference proceedings from general and speciality meetings. This is an ongoing activity across the Cochrane Collaboration and is both retrospective and prospective (Master List 2007, http://apps1.jhsph.edu/cochrane/masterlist.asp). Therefore we will not specifically search conference proceedings. Please refer to The Cochrane Renal Group's Module in *The Cochrane Library *for the most up-to-date list of conference proceedings (Renal Group 2008).

2. MEDLINE (1966 to July 2009) using the optimally sensitive strategy developed for the Cochrane Collaboration for the identification of RCTs ([27]) together with a search strategy developed with input from the Cochrane Renal Group's Trial Search Co-ordinator.

3. EMBASE (1980 to July 2009) using a search strategy adapted from that developed for the Cochrane Collaboration for the identification of RCTs ([28]) together with the a search strategy developed with input from the Cochrane Renal Group's Trial Search Co-ordinator.

4. Reference lists of nephrology textbooks, review articles and relevant studies.

5. Letters seeking information about unpublished or incomplete studies to investigators known to be involved in previous studies. Letters seeking information about unpublished or incomplete studies to investigators known to be involved in previous studies.

Database Search terms

#1 wegener*

#2 (rapid* near progress* near glomeruloneph*) or (crescent* near glomeruloneph*)

#3 glomerulonephritis

#4 (antineutrophil near cytoplasmic)

CENTRAL   #5 vasculitis

#6 goodpasture

#7 polyarteritis

#8 (acute near glomeruloneph*)

#9 (#1 or #2 or #3 or #4 or #5 or #6 or #7 or #8)

1. exp WEGENER'S GRANULOMATOSIS/

2. exp Antibodies, Antineutrophil Cytoplasmic/or exp Vasculitis/or exp Glomerulonephritis/or exp Polyarteritis Nodosa/

3. rapidly progressive glomerulonephritis.ti.

4. exp GOODPASTURE SYNDROME/

5. vasculitis.tw. (12821)

6. polyarteritis.tw. (1928)

7. wegener$.tw. (3673)

8. (rapid$ adj25 progress$ adj25 glomeruloneph$).ti,ab. (906)

9. or/1-8 (86982)

10. randomized controlled trial.pt. (189812)

11. controlled clinical trial.pt. (66435)

12. randomized controlled trials/(32645)

13. random allocation/(50890)

14. double blind method/(78368)

15. single blind method/(8139)

MEDLINE   16. or/10-15 (321286)

17. animals/not (animals/and human/) (2792757)

18. 16 not 17 (304657)

19. clinical trial.pt. (383960)

20. exp clinical trials/(155138)

21.(clinic$ adj25 trial$).ti,ab. (99992)

22. cross-over studies/(14545)

23. (crossover or cross-over or cross over).tw. (32414)

24. ((singl$ or doubl$ or trebl$ or tripl$) adj25 (blind$ or mask$)).ti,ab. (77495)

25. placebos/(23040)

26. placebo$.ti,ab. (84360)

27. random$.ti,ab. (285800)

28. research design/(38137)

29. or/19-28 (683077)

30. 29 not 17 (633481)

31. 18 or 30 (643563)

32. 9 and 31 (3934)

1. exp wegener's granulomatosis/or wegener's granulomatosis.mp.

2. exp Rapidly progressive glomerulonephritis/or rapidly progressive glomerulonephritis.mp.

3. exp neutrophil cytoplasmic antibody/or neutrophil cytoplasmic antibody.mp.

4. exp vasculitis/or vasculitis.mp.

5. exp goodpasture syndrome/or goodpasture syndrome.mp.

6. exp polyarteritis nodosa/or polyarteritis nodosa.mp.

7. exp acute glomerulonephritis/or acute glomerulonephritis.mp.

8. exp proliferative glomerulonephritis/or proliferative glomerulonephritis.mp.

9. exp glomerular basement membrane antibody/or glomerular basement membrane antibody.mp.

10. exp glomerular basement membrane/or glomerular basement membrane.mp.

11. or/1-10

12. exp clinical trial/

13. evidence based medicine/

14. outcomes research/

15. crossover procedure/

EMBASE   16. double blind procedure/

17. single blind procedure/

18. prospective study/

19. exp comparative study/

20. placebo/

21. "evaluation and follow up"/

22. follow up/

23. randomization/

24. or/12-23

25. controlled study/not case control study/

26. or/24-25

27. (clinic$ adj5 trial$).ti,ab.

28. ((singl$ or doubl$ or trebl$ or tripl$) adj (blind$ or mask$)).ti,ab.

29. random$.ti,ab.

30. placebo$.ti,ab.

31. or/27-30

32. or/24,31

33. limit 32 to human

34. 11 and 33

## Pre-publication history

The pre-publication history for this paper can be accessed here:

http://www.biomedcentral.com/1471-2369/11/12/prepub
